# Examination of the Microbial Spectrum in the Etiology of Erythema Nodosum: A Retrospective Descriptive Study

**DOI:** 10.1155/2017/8139591

**Published:** 2017-05-28

**Authors:** Ozlem Ozbagcivan, Sevgi Akarsu, Ceylan Avci, Burcu Bahar Inci, Emel Fetil

**Affiliations:** ^1^Department of Dermatology, Faculty of Medicine, Dokuz Eylul University, Izmir, Turkey; ^2^Department of Dermatology, Bilecik State Hospital, Bilecik, Turkey

## Abstract

Even though infections are the most common cause of erythema nodosum (EN), only certain microorganisms take the great interest such as streptococci in knowledge. Our aim was to examine the frequency and type of infections in EN, to determine the characteristics of patients with an infectious etiology, and to discuss the role of these microbes in EN pathology in the context of their interactions with humans. Charts of 81 patients with EN who were seen between 2003 and 2017 were retrospectively reviewed. Identified etiological factors were classified into three groups: infectious, noninfectious, and idiopathic. While there were no significant demographic and clinical differences between the infectious and idiopathic groups, systemic symptoms (*p* = 0.034) and the number of EN lesions (*p* = 0.016) were significantly lower; the mean erythrocyte sedimentation rate was significantly higher (*p* = 0.049), but the mean aspartate aminotransferase value was significantly lower in the infectious group compared to the noninfectious group (*p* = 0.019). Besides streptococci, many other microbes, including the ones living on and inside us, were identified in the etiology of EN. There is a need for large-scale prospective studies involving control groups for a better understanding of the microbial immunopathology of EN.

## 1. Introduction

Erythema nodosum (EN) is the most common clinical form of panniculitis defined with hypodermal septal inflammation. It usually appears on the anterior surfaces of the lower extremities as erythematous, warm, and painful nodules ranging from 1 to 20 cm in diameter [1]. Lesions do not have a tendency to necrosis and scars but may leave residual hyperpigmentation. Cutaneous symptoms reach their maximum in 1-2 weeks and then spontaneously resolve in 1–6 weeks, sometimes taking up to 12 weeks to fully resolve [2, 3]. In addition to the cutaneous symptoms, a prodrome commonly occurs a few weeks before the onset of EN including weight loss, malaise, low-grade fever, cough, and arthralgia with or without arthritis [1, 2, 4].

The exact prevalence of EN in Turkey is unknown, but the estimated worldwide prevalence of the disease is reported as one to five per 100,000 persons [2, 5]. Although EN can occur at any age, it is most common in the third and fourth decades of the life, and there is a female predominance with the prevalence being three to five times higher in women than men [3].

EN is generally considered a reactive process that may be triggered by a wide variety of stimuli [1, 2]. Most direct and indirect evidence support the involvement of a type IV delayed hypersensitivity response to numerous antigens. Although no specific cause can be documented in most of the patients, it is imperative to investigate the possible triggers [3]. Etiological factors may vary according to many conditions including age, gender, race, and geographic location of the patient, but infections are the most frequently related factors [1, 2, 6]. Because of the well-known association of EN with streptococcal infections, they take the great part of interest in most studies; however, other infections are not sufficiently focused in this regard. Therefore, there is a need to think about the possible roles and locations of other microorganisms in the etiology of EN.

The purpose of this study was to examine the frequency and type of infections in EN etiology, to determine the characteristics of patients with an infectious etiology and to discuss the role of these microbes in EN pathology in the context of their interactions with humans.

## 2. Materials and Methods

### 2.1. Study Design

This is a retrospective chart review of all patients with EN who were seen at the Department of Dermatology of Dokuz Eylul University Faculty of Medicine in Izmir, Turkey, between January 2003 and 2017. The study protocol was approved by the Local Ethical Committee which follows the guidelines set by the Declaration of Helsinki.

### 2.2. Collection of the Data

A total of the 159 patients' file records with the diagnosis of EN were screened. The diagnosis was made on clinical grounds based on the presence of tender erythematous nodules and plaques without ulceration mainly on the legs. Skin biopsies were carried out in 64 patients with unilateral involvement and in those with involvement at sites other than the pretibial areas. Histopathological verification was based on the presence of septal inflammation, with an inflammation of the septal vessels, but without signs of true vasculitis. 18 patients were excluded because of the absence of typical histopathological findings, and 60 patients were excluded because of the missing data, lack of ≥1-year follow-up period in their file records, and having more than single etiological factor. As a result, a total of 81 patients were included in this study, and 46 of them were verified by histopathologic examination due to the clinical suspicion. Demographic and clinical data were collected from patients' file records. Clinical features including systemic manifestations, location and number of EN lesions, past history of EN, season in the EN attack, associated disorders, and used medications were noted. The following investigations were done in all included patients: complete blood count; erythrocyte sedimentation rate; C-reactive protein; routine blood chemistry including fasting blood glucose, urea, creatinine, aspartate aminotransferase, alanine aminotransferase, alkaline phosphatase, gamma glutamyl transferase, lactate dehydrogenase, total bilirubin, direct bilirubin, and total protein; serum electrolytes including sodium, potassium, chloride, and calcium; viral serologies including human immunodeficiency virus, hepatitis B virus, and hepatitis C virus; serologies for hydatid cyst, brucella, and syphilis; antistreptolysin O titer (two consecutive determinations were performed in a 2–4 week interval if it was >200 U/mL); antinuclear antibody; routine urine analysis; urine culture; throat culture; direct fecal smear microscopy and culture; tuberculin skin test; chest X-ray; and abdominopelvic ultrasonography. In each case, the results of routinely requested dental, otorhinolaryngological, and gynecological consultations were also reviewed in order to investigate focal infections. Vaginal smears and cultures were obtained from all patients who underwent vaginal examination. Specific investigations such as angiotensin converting enzyme, pathergy test, computed tomography scan, and special microbial smears, cultures, and/or serologies were also performed in individual patients if there were associated signs and symptoms.

### 2.3. Etiological Classification of EN

EN etiology was classified into three groups: infectious (associated with any infection), noninfectious (associated with rheumatological diseases, sarcoidosis, inflammatory bowel diseases, malignancies, and/or medications), and idiopathic (when no underlying disease or precipitating event was found). Criteria for including the patients in the infectious group were the absence of any other identifiable factors and detection of no recurrences of EN within 1 year after the appropriate treatment of the identified infection. In order to provide a clear distinction in the classification, only the patients who had only single well-established etiological cause were included in this study. In other words, patients who had both infectious and noninfectious etiologies were excluded.

### 2.4. Statistical Analysis

The statistical analyses were performed with the SPSS/PC software (Version 23.0 for Windows; SPSS Inc., Chicago, Ill). The Mann Whitney *U* test was used to compare the mean values of quantitative variables as the two samples were obtained independently. Qualitative variables were analyzed with chi-squared test and Fisher's exact test. *p* < 0.05 was considered significant in all analyses.

## 3. Results

Demographic and clinical characteristics of the patients with EN are shown in Table 1. Overall, the most common etiological factor was infections which were found in 32 (39.5%) of the patients (Table 2). Among all patients with EN, 19 (23.4%) had recent upper respiratory tract infections and 13 (16%) of them had serological evidence for streptococcal pharyngitis (a positive throat swab culture for *Streptococcus pyogenes* in one patient and high antistreptolysin O titers in the others). All infections and identified microbes in EN patients are demonstrated in Table 3.

In the comparison of three groups (infectious, noninfectious, and idiopathic), no significant differences were observed between the infectious and idiopathic groups in means of all demographic and clinical data. But, while infectious and noninfectious groups did not show significant differences in terms of age, gender, past EN history, location of EN lesions, and season in the EN attack, the presence of systemic symptoms and number of EN lesions were significantly lower in the infectious group (*p* = 0.034 and *p* = 0.016, resp.). In a detailed examination of the systemic symptoms, even though they did not reach statistical significance, malaise and arthralgia were the markedly lower symptoms in the infectious group (Table 4). In the infectious group, a majority of patients had the EN attack in autumn (34.4%) while the number of patients were the lowest in autumn for the other two groups. However, these differences did not show statistical significance.

**Figure 1:**
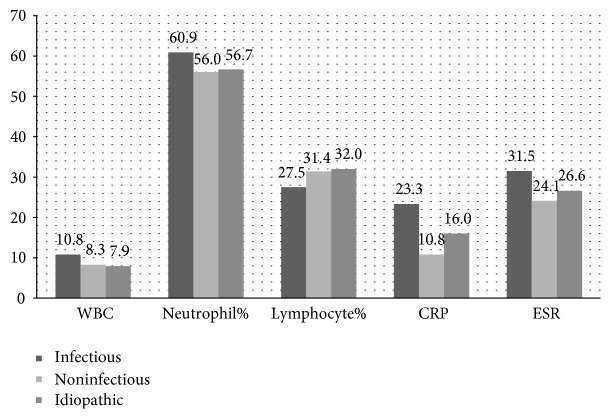
Laboratory parameters associated with infectious processes in erythema nodosum patients according to the etiological classification (*n* = 81). WBC: white blood cell; CRP: C-reactive protein; ESR: erythrocyte sedimentation rate.

In laboratory evaluations, higher white blood cell count, neutrophil percentage, C-reactive protein value, and an erythrocyte sedimentation rate were found in the infectious group compared to the other groups (Figure 1). Among these, only the differences in the erythrocyte sedimentation rate (31.5 mm/h versus 24.1 mm/h) and in the proportion of patients with the high erythrocyte sedimentation rate (65.6% versus 33.3%) showed statistical significance between the infectious and noninfectious groups (*p* = 0.049 and, resp.). By contrast, many of the biochemical markers were found to be higher in the noninfectious group compared to the infectious group. However, only the difference in the proportion of patients with high aspartate aminotransferase (0% versus 16.7%) reached the statistical significance (*p* = 0.029) (Table 5).

## 4. Discussion

In this report, we present data based on the spectrum of the microbial etiology of EN in a series of patients seen during a 14-year period in a university hospital for a defined population of Western Turkey. Not as a surprising result, the etiology of EN was associated with infectious causes in a high proportion of patients, and streptococci were the most frequently identified etiological agents. But the main outcome of this study is that, besides the well-known associations of certain microbes with EN, even some others that we consider to be harmless or microorganisms which do not constitute the criteria for an active infection, may also involve in EN etiology.

In addition, to examine the microbial diversity in EN etiology, we also aimed to determine the specific characteristics of patients with an infectious etiology. We found that systemic symptoms and the number of EN lesions were lower in these patients when compared to the patients with a noninfectious etiology. Since most of the patients with an infectious etiology had no current active infection or their infections were in the regressive phase at the time when EN lesions were detected, systemic symptoms may also be reduced in this period. Another reasonable explanation of these results may be the detection of some silent infections in these patients such as *Blastocystis hominis* (*B. hominis*), *Helicobacter pylori* (*H. pylori*), *Gardnerella vaginalis* (*G. vaginalis*), or *Candida albicans* (*C. albicans*). Additionally, the higher detection of the long standing symptoms such as weight loss, malaise, arthralgia, and night sweats in the other group is already an expected finding as a result of the course of the chronic systemic diseases. In laboratory findings, a significantly higher erythrocyte sedimentation rate in patients with an infectious etiology may be reflecting the slow regression of this acute phase reactant which might be increased in the active phase of the infections. On the other hand, significantly higher aspartate aminotransferase values in the noninfectious group may be related with the accompanying systemic disorders in these patients. Although this enzyme is basically known as a diagnostic liver enzyme, various conditions may increase the value since it is located in many tissues of the human body. Two of the patients with high aspartate aminotransferase values in the noninfectious group had malignancies (pancreas cancer in one patient and myelodysplastic syndrome in the other), one of the patients had systemic sarcoidosis, and the other one had long-term oral contraceptive use, which may be the factors responsible for the high values in patients in this study.

The spectrum of the microbial etiology of EN may vary depending on a range of factors such as the population studied, the location, reflecting the geographical distribution of microbes, and even the department in the same geographical area in which patients were admitted [1, 2, 5]. Although all the infective agents including aerobic and anaerobic bacteria, viruses, fungi, parasites, and mycobacteria can induce eruption of EN, a specific cause can be documented in only half of the patients in clinical practice. Streptococcal pharyngitis is still the most frequent etiological factor in all over the world both in developed and developing countries [7–10]. They have been reported to be detected up to 44% of patients in adults and 48% of patients in children with EN [4]. The increased incidence of EN patients in the first half of the calendar year and the detection of familial EN cases may also be associated with this infectious etiology [1]. EN eruptions typically appear two to three weeks after the episode of a streptococcal pharyngitis so that the pharyngeal swabs are usually negative at the time when EN lesions are detected, as in our cases. Therefore, patients should have repeated streptococcal antistreptolysin O titers or polymerase chain reaction assays for group A streptococci to demonstrate the previous infection [4].

Apart from upper respiratory tract infections, some gastrointestinal system infections were also identified as the etiological factor in this study. Intestinal *B. hominis* infection was determined in stool in two of the patients with intermittent abdominal pain and diarrhea. *B. hominis* is an intestinal protozoan regarded by some as a mere commensal and by others as a parasite that can cause diarrhea and intestinal disease [11]. Although there has been no documentation in literature to specify the association of *B. hominis* with EN, it has been known that some intestinal parasites such as *Giardia* species have been reported to be associated with EN in literature [12, 13]. Furthermore, *B. hominis* has already been known to cause cutaneous hypersensitivity reactions by alternating the immune system via activation of specific Th2 immune cells producing interleukins including IL-3, IL-4, IL-5, and IL-13, and some immunological disorders such as urticaria, Hashimoto's thyroiditis, and Henoch-Schönlein purpura have been mentioned to be associated with *B. hominis* infection [11, 14, 15]. While all human intestinal parasitic infections have been shown to induce both humoral and cellular immune responses and can cause the hypersensitivity reactions in their hosts, we suggest a possible role of *B. hominis* infection in the pathogenesis of EN.

The occurrence of EN lesions was associated with *H. pylori* infection of the stomach in another patient in this study. Both stool antigen assay and endoscopic biopsy were performed as the diagnostic methods for this infection. *H. pylori* is the dominant member of the gastric microbiota in over half of the human population, and it can persist in a human stomach for a lifetime. Approximately 20% of *H. pylori* organisms adhere to the surface of gastric epithelial cells, and this physical contact causes cellular damage and activates the immune system [16]. Since this immunological response caused by the bacterium is oriented both locally and systemically, several immune-mediated extraintestinal disorders including hematological, cardiovascular, neurological, metabolic, and autoimmune have been considered to be potentially induced by the infection. Even though the presence of reports in literature on the association of *H. pylori* with various cutaneous diseases such as chronic urticaria, rosacea, psoriasis, Henoch-Schönlein purpura, Behçet's disease, alopecia areata, and Sweet syndrome, there exists only one report to demonstrate the association with EN [17]. In this short report of Capella GL. consisting of two patients, the author discussed the possible role of *H. pylori* infection in the etiology of EN by providing some scientific evidence. These were its biological similarity with *Campylobacter* species which are the recognized causes of EN, the presence of EN-like eruptions in immunosuppressed patients caused by *Helicobacter cinaedi* bacteremia, the disappearance of EN-like lesions of Behçet's disease after *H. pylori* eradication, and the improvement of EN lesions after *H. pylori* eradication [18].

Urinary tract infection due to *Escherichia coli* (*E. coli*) was the associated factor with EN lesions in three of the patients in our study. *E. coli* is one of the most versatile bacterial species which is either harmless as a commensal in gut microbiata or pathogenic with the ability to cause intestinal or extraintestinal infections [19]. It is becoming increasingly clear that various microbes have been postulated to trigger a cascade of immunological events in patients, and urinary tract infections are among the most common infectious diseases of humans caused by a range of pathogens, with uropathogenic *E. coli* being the most common etiological agent [20]. Even with this high frequency, the association of *E. coli* infections with EN has not been adequately addressed in literature. In only one study, investigating the associated diseases with EN, *E. coli* infection was mentioned among the well-established associations. However, the authors gave no detailed information about the type or anatomical location of the infection in this study [7].

Two of the study patients had EN lesions associated with pneumonia in our study. An empiric antibiotic therapy was started without the ability to determine the infectious agent in one patient, and *Mycoplasma pneumoniae* (*M. pneumoniae*) was the identified agent in the other. There are many publications in literature reporting the association between *M. pneumoniae* and EN [21, 22]. Unfortunately, the link between EN and mycoplasma infection has not been fully understood yet. In some instances, *M. pneumoniae* has been isolated directly from skin lesions, suggesting the direct effect of the microorganism to elicit the development of cutaneous lesions [23]. It has been speculated that *M. pneumoniae* can be transferred hematogenously to the dermis, thus causing a hypodermal inflammation. However, it is also impossible to exclude the role of immunologic mechanisms and autoimmunity [21] because most cutaneous lesions associated with microorganisms are thought to be caused not by the organisms themselves but by the host response to antigens on these microbes. Many different mechanisms such as immune-complex-mediated damage, cytotoxic T cell-mediated immune responses, cytokines and chemokines released against mycoplasmal lipopeptides, and autoimmune reactions may play a role in some phases of the immune responses, and the innate immune response to mycoplasmas may also enhance the role of the adaptive immunity [23].

Genital infections were detected in two of the EN patients in this study. The first one was bacterial vaginosis caused by *G. vaginalis*. Bacterial vaginosis is the worldwide leading vaginal disorder among women of reproductive age caused by polymicrobial agents in which *G. vaginalis* is the founder organism [24]. However, *G. vaginalis* is also considered to be a part of the normal vaginal microbiota in a significant proportion of both girls and women by some researchers while others think of this entity as the asymptomatic infection [25]. When we consider the association of this microbe with some immunological conditions such as erythema multiforme and reactive arthritis, it may not be irrational to consider it among the microbial etiological causes of EN [26, 27]. The other detected genital infection was vulvovaginal candidiasis caused by *C. albicans* in the second patient. Vulvovaginal candidiasis is estimated to be the second most common cause of vaginitis after bacterial vaginosis, and *C. albicans* accounts for 85% to 90% of cases [28]. *C. albicans* is a commensal yeast species that is found in the human gastrointestinal and vaginal microbiota. When tolerance mechanisms become defective, the commensal form changes into the pathogenic form and expresses its virulence traits [29]. In our literature search, we found no specific documentation on the association of *C. albicans* with EN, although the delayed-type hypersensitivity reaction to *C. albicans* is already well known and has been historically used to evaluate the immune competence experimentally in humans [30]. In fact, the essential subject that might be considered in this aspect is whether these microorganisms are among the overlooked etiological factors in EN cases in which there exists a female predominance, especially in patients who fall into the “idiopathic” category.

Breast abscesses are among the relatively rare reported causes of EN in literature. In a patient series involving two EN patients associated with breast abscesses, *Staphylococcus aureus (S. aureus*) was the causative agent in one of the patients as compatible with our results [31]. In spite of the pathogenesis associated with *S. aureus*, it is also acknowledged to be a typical member of the complex community of microbiota living on and within our skin. But little is known about the transition from the asymptomatic colonization to an invasive infection, despite the fact that persistently colonized individuals are most often infected by their own colonizing strains [32]. Although *S. aureus* is the most frequently detected microorganism in breast abscesses, the relationship with EN is still unclear due to the low level of evidence [31].

Tuberculosis has long been linked with EN and is the second most common infectious disease associated with EN after streptococcal infections in most reports from Turkey [6, 33]. EN may occur in the course of primary tuberculosis and may even manifest before the development of a skin reaction to the tuberculin skin test. Furthermore, EN may be found in patients with highly positive reactions to the tuberculin skin test with no detectable focus of active tuberculosis infection. It is believed to reflect the strong immunologic response to *Mycobacterium tuberculosis* (*M. tuberculosis*) antigens in immunocompetent individuals [4, 34]. Consistent with this, an excess cytokine response to *M. tuberculosis* antigens was demonstrated in EN patients in a recent study, and blood from EN patients was shown to exhibit an enhanced ability to restrict the mycobacterial growth in vitro. On the other hand, in some studies, it was suggested that EN should be considered a strong predictor of tuberculosis or an early symptom of tuberculosis rather than just a response to newly acquired *M. tuberculosis* antigens. It has been suggested to screen all patients with EN for *M. tuberculosis* infection and even to initiate the tuberculosis treatment in certain patients who are in hard-to-reach populations with limited possibility of continuous monitoring for the symptoms [34]. In our study, contrary to the expected for our country, tuberculosis did not constitute a major portion of the EN etiology. The reason for this may be the lower incidence of tuberculosis in our region compared to the other regions of the country [35].

The major limitations of this study are the retrospective nature, lack of a control group to compare the results obtained, and limited number of patients due to our intention to take the patients only who had complete data and single well-established etiological factor for inclusion. Additionally, the follow-up periods were not uniform in each case; thus, it may have caused some missing follow-up data for the detection of recurrences of EN in patients. However, as far as we know, this is the first detailed study in literature that examines and discusses the possible roles of microbes in EN occurrence, in the context of their interactions with humans.

## 5. Conclusion

In the present study, a significant proportion of cases with EN was associated with the infectious etiology, and streptococcal pharyngitis was the most commonly detected cause in accordance with previous reports. Besides this well-known association, *B. hominis*, *H. pylori*, *E. coli*, *M. pneumoniae*, *G. vaginalis*, *C. albicans*, *S. aureus*, and *M. tuberculosis* were the other identified microbes in EN development in our patients. Since all microbes, either pathogenic or commensal, are the critical regulators of the host immune system and have the potential to induce the activation of immunological events in their hosts through complex mechanisms, the microbial spectrum of EN may be much greater than we know. There is a need for prospective large-scale studies involving control groups in the future for a better understanding of the microbial immunopathology of EN.

## Figures and Tables

**Table 1:** Demographic and clinical characteristics of the study subjects (*n* = 81).

Characteristics	EN patients
Gender, *n* (%)	
Female	64 (79%)
Male	17 (21%)
Age, mean ± SD (min–max)	38.54 ± 19.17 (7–83)
Location of EN	
Pretibial areas	59 (72.8%)
Pretibial areas + other areas^∗∗^	14 (17.3%)
Other areas^∗∗^	8 (9.9%)

EN: erythema nodosum; ^∗∗^femoral areas, gluteal areas, arms, ankles, and/or knees.

**Table 2:** Etiological factors in patients with erythema nodosum (*n* = 81).

Etiological factors	*n* (%)
Infectious	32 (39.5%)
Noninfectious	24 (29.6%)
Rheumatological diseases	11 (13.5%)
Sarcoidosis	6 (7.4%)
Inflammatory bowel diseases	(3.7%)
Malignancies	2 (2.5%)
Medications	2 (2.5%)
Idiopathic	25 (30.9%)

**Table 3:** Infections and identified microbes in patients with erythema nodosum (*n* = 81).

Infections	Identified microbes	Patients with EN
URTI		19 (23.4%)
Streptococcal	Streptococci (*n* = 13)	13 (16%)
Nonstreptococcal	*—*	3 (3.7%)
Viral	*—*	3 (3.7%)
GIT infection		3 (3.7%)
Intestinal infection	*Blastocystis hominis* (*n* = 2)	2 (2.5%)
Stomach infection	*Helicobacter pylori* (*n* = 1)	1 (1.2%)
UTI	*Escherichia coli* (*n* = 3)	3 (3.7%)
Pneumonia	*Mycoplasma pneumoniae* (*n* = 1)	2 (2.5%)
Vaginitis	*Gardnerella vaginalis* (*n* = 1) *Candida albicans* (*n* = 1)	2 (2.5)
Breast abscess	*Staphylococcus aureus* (*n* = 1)	1 (1.2%)
Rectal abscess	*—*	1 (1.2%)
Tuberculosis	*Mycobacterium tuberculosis* (*n* = 1)	1 (1.2%)

EN: erythema nodosum; URTI: upper respiratory tract infection; GIT: gastrointestinal tract; UTI: urinary tract infection.

**Table 4:** Demographic and clinical data of erythema nodosum patients according to the etiological classification (*n* = 81).

Variables	Etiological classification			*p* value	
	Infectious (A)	Noninfectious (B)	Idiopathic (C)	A versus B	A versus C
Number of patients, *n* (%)	32 (39.5)	24 (29.6)	25 (30.9)	—	—
Age, years, mean ± SD	36.16 ± 18.17	43.88 ± 17.39	36.48 ± 21.65	0.110	0.987
Female gender, *n* (%)	26 (81.3)	18 (75)	20 (80)	0.573	1.00
Systemic symptoms, *n* (%)	15 (46.9)	18 (75)	6 (24)	**0.034** ^∗^	0.076
Fever	6 (18.8)	5 (20.8)	2 (8)	1.00	0.444
Weight loss	2 (6.3)	4 (16.7)	1 (4)	0.385	1.00
Malaise	2 (6.3)	6 (25)	0	0.063	0.499
Arthralgia	10 (31.3)	13 (54.2)	5 (20)	0.085	0.339
Night sweats	1 (3.1)	2 (8.3)	0	0.571	1.00
Number of EN lesions, mean ± SD	1.90 ± 0.92	2.95 ± 1.82	2.24 ± 1.47	**0.016** ^∗^	0.603
Past history of EN, *n* (%)	3 (9.4)	5 (20.8)	5 (20)	0.268	0.280
Location of EN, *n* (%)					
Pretibial areas	25 (78.1)	17 (70.8)	17 (68)	0.533	0.389
Pretibial areas + other areas^∗∗^	4 (12.5)	5 (20.8)	5 (20)	0.475	0.485
Other areas^∗∗^	3 (9.4)	2 (8.3)	3 (12)	1.00	1.00
Season in the EN attack, *n* (%)					
Spring	5 (15.6)	7 (29.2)	6 (24)	0.222	0.508
Summer	8 (25)	7 (29.2)	8 (32)	0.728	0.559
Autumn	11 (34.4)	3 (12.5)	5 (20)	0.061	0.231
Winter	8 (25)	7 (29.2)	6 (24)	0.728	0.931

EN: erythema nodosum. ^∗^Significant values. ^∗∗^Femoral areas, gluteal areas, arms, ankles, and/or knees.

**Table 5:** Laboratory characteristics of erythema nodosum patients according to the etiological classification (*n* = 81).

Variables	Etiological classification			*p* value	
	Infectious (A)	Noninfectious (B)	Idiopathic (C)	A versus B	A versus C
	(*n* = 32)	(*n* = 24)	(*n* = 25)		
*Complete blood count*					
WBC > 10,000/mm^3^, *n* (%)	11 (34.4)	6 (25)	5 (20)	0.450	0.231
Neutrophil > 73%, *n* (%)	5 (15.6)	1 (4.2)	1 (4)	0.085	0.215
Lymphocyte > 45%, *n* (%)	2 (6.3)	4 (16.7)	3 (12)	0.385	0.645
*Acute phase reactants*					
CRP > 5 mg/L, *n* (%)	18 (56.3)	11 (45.8)	10 (40)	0.440	0.223
ESR > 20 mm/h, *n* (%)	21 (65.6)	8 (33.3)	12 (48)	**0.017** ^∗^	0.181
*Biochemical markers*					
FBG > 100 mg/dL, *n* (%)	8 (25)	6 (25)	5 (20)	1.00	0.655
Urea > 20 mg/dL, *n* (%)	0	1 (4.2)	0	0.429	NA
Creatinine > 0.95 mg/dL, *n* (%)	1 (3.1)	3 (12.5)	1 (4)	0.303	0.439
AST > 35 U/L, *n* (%)	0	4 (16.7)	1 (4)	**0.029** ^∗^	0.439
ALT > 35 U/L, *n* (%)	1 (3.1)	5 (20.8)	1 (4)	0.074	1.00
LDH > 220 U/L, *n* (%)	10 (31.3)	8 (33.3)	8 (32)	1.00	1.00
GGT > 38 U/L, *n* (%)	3 (9.4)	5 (20.8)	2 (8)	0.268	1.00
ALP > 120 U/L, *n* (%)	3 (9.4)	4 (16.7)	3 (12)	0.447	1.00
Total bilirubin > 1.2 mg/dL, *n* (%)	0	1 (4.2)	0	0.429	NA
Direct bilirubin > 0.2 mg/dL, *n* (%)	3 (9.4)	5 (20.8)	4 (16)	0.268	0.687
Total protein < 8.3 g/dL, *n* (%)	4 (12.5)	5 (20.8)	3 (12)	0.475	1.00

WBC: white blood count; CRP: C-reactive protein; ESR: erythrocyte sedimentation rate; FBG: fasting blood glucose; AST: aspartate aminotransferase; ALT: alanine aminotransferase; LDH: lactate dehydrogenase; GGT: gamma glutamyl transferase; ALP: alkaline phosphatase; NA: not applicable. ^∗^Significant values. (The upper or lower limits in laboratory values were based on the reference intervals given in Central Laboratory of Dokuz Eylul University Hospital.)
